# Successful Treatment of SCD-Related Priapism With Crizanlizumab: A Case Series

**DOI:** 10.1177/23247096231191873

**Published:** 2023-09-20

**Authors:** Modupe Idowu, Roberto L. Garcia, Oluwatosin B. Sule

**Affiliations:** 1 Department of Internal Medicine, John P. and Kathrine G. McGovern Medical School, The University of Texas Health Science Center at Houston, Houston, TX, USA

**Keywords:** sickle cell disease, priapism, crizanlizumab

## Abstract

Priapism, an unwanted, painful, prolonged erection that is unrelated to sexual stimulation, is a common complication of sickle cell disease (SCD). Priapic events in SCD are stuttering, meaning they occur repeatedly with intervening periods of detumescence. Without health care intervention, repeated episodes can lead to erectile dysfunction. There are limited treatment options for SCD-related priapism and no approved targeted therapies. Crizanlizumab is a monoclonal antibody that binds to P-selectin and is used to reduce the frequency of vaso-occlusive crises in patients with SCD. Here, we report the cases of 3 patients with SCD-related priapism who were treated with crizanlizumab. All patients were African American men who experienced numerous priapic episodes that interfered with their daily lives. Upon treatment with crizanlizumab, priapic events were reduced in all 3 patients. These successful cases suggest a potential role for crizanlizumab in the prevention of SCD-related priapism.

## Introduction

Sickle cell disease (SCD) is a systemic condition and one of the most common heritable hematologic diseases, affecting many worldwide.^1^ Sickle cell disease causes hemoglobin polymerization that distorts red blood cells (RBCs) into a sickle shape. These sickle RBCs can aggregate and block narrow capillaries, causing painful events known as vaso-occlusive crises (VOCs) and organ damage.^1^ Priapism is an unwanted protracted erection that may last several hours and is a complication experienced by many males with SCD. Sickle cell disease–related priapism is not only due to sickle RBCs blocking vascular channels in the penis but also through vasoconstriction caused by hemolysis, reduced nitric oxide (NO) availability, and aberrant cyclic guanosine monophosphate signaling.^1^

Ischemic priapism, also known as low-flow or veno-occlusive, accounts for >95% of cases and involves venous occlusion with restricted arterial blood flow to the corpus cavernosum, leading to rigidity and pain similar to compartment syndrome.^1,2^ Ischemic priapic events lasting >4 hours warrant urgent medical treatment because prolonged events can cause tissue necrosis and irreversible damage.^2,3^ American Urological Association guidelines recommend corporal aspiration, then intracavernosal injection of sympathomimetics, followed by surgical shunting and insertion of penile prostheses as therapies for ischemic priapism.^2^

Stuttering priapism is a subtype of ischemic priapism characterized by painful events that last from a few minutes to several hours and occur repeatedly with intervening periods of detumescence.^1,3^ Sickle cell disease is the most common cause of stuttering priapism, affecting approximately 40% of adult men and 65% of children and adolescent boys with SCD.^3,4^ Stuttering priapic events are generally self-limiting; however, repeated episodes can lead to permanent damage and erectile dysfunction.^3^ Priapic events account for significant health care resource utilization and impaired quality of life due to diminished sexual function, sleep disorders, and psychological stress.^4^

Initial management of SCD-related priapism is usually done at home; however, approximately one third of cases develop into ischemic priapism and require urgent medical care.^1,3^ Urologic consultation should be obtained emergently to evaluate patients for possible aspiration and irrigation.^1^ Pharmacological treatments are limited because none are approved for the management of priapism, and few studies on efficacy exist.^1^ Evidence supporting the use of RBC transfusion is lacking, and reports of associated neurological complications have been published.^1^

This case series presents 3 patients with SCD-related priapism who were successfully treated with crizanlizumab. Crizanlizumab is a monoclonal antibody that binds to P-selectin, blocking its interaction with glycoprotein ligand 1, which has been shown to play a pathogenic role in VOCs in SCD.^5^ Crizanlizumab is currently under investigation in a phase 2, open-label, single-arm, multicenter study to assess efficacy and safety in patients with SCD-related priapism (NCT03938454).^6^

## Results

### Patient 1

Patient 1 is a 26-year-old African American man with a history of SCD (HbSS), priapism, iron-deficiency anemia, and 1 or 2 VOCs per year that required hospital admission (Table 1). He was receiving hydroxyurea for the treatment of SCD but discontinued in 2020 due to his inability to afford the medication.

**Table 1.** Patient Medical History and Laboratory Results.

	Patient 1	Patient 2	Patient 3
Medical history	• SCA HbSS • Iron-deficiency anemia • Priapism	• SCA HbSS • Priapism • Pneumonia • Pulmonary embolism • Thrombocytosis	• SCA HbS/β^0^-thalassemia • Two cases of pneumonia, with last episode in January 2019 • Jaundice
Family history	**Both parents**: sickle cell trait **Brother:** sickle cell disease	**Both parents:** sickle cell trait **Sibling:** sickle cell trait	**Grandmother:** diabetes
Medications	Folic acid 1 mg daily • Hydrocodone/acetaminophen 10-325 mg every 6 hours as needed • Ibuprofen 600 mg every 8 hours as needed	Folic acid 1 mg daily • Hydroxyurea 1500 mg daily • Hydrocodone/acetaminophen 10-325 mg every 6 hours as needed	Folic acid 1 mg daily
Hb level, g/dL	9.8	7.2	9.9
Reticulocytes, %	5.9	6.3	6.4
LDH, IU/L	Hemolyzed	331	504
Total bilirubin, mg/dL	3.6	1.4	2.9
Creatinine, mg/dL	0.7	0.6	0.6
Blood pressure, mm Hg	131/73	102/63	113/67
Heart rate, bpm	63	72	61

Abbreviations: SCA, sickle cell anemia; β^0^, beta null; Hb, hemoglobin; LDH, lactate dehydrogenase.

Patient 1 first experienced priapism in 2016, which progressed to nightly episodes from 12:00 am to 3:00 am that lasted 1 to 2 hours. Symptoms included a sharp stomach pain followed by a prolonged erection. Self-care at home included hot showers, walking, or playing video games to distract from symptoms. Within a few hours, the priapic events resolved but reoccurred the next night. While the patient was advised to go to the emergency department for events lasting >3 hours, he expressed hesitancy because of the potential for an aspiration procedure.

In November 2021, the patient received his first crizanlizumab infusion at a dose of 5 mg/kg, with a subsequent infusion 2 weeks later and every 4 weeks thereafter. During the first infusion, he began to experience symptoms consistent with a pain episode. This infusion-related reaction (IRR) was managed with intravenous (IV) fluids at the infusion clinic followed by further management at home. The following night, the patient did not experience any priapic events and has not had any since starting crizanlizumab. The patient qualitatively self-reported improved quality of life since initiating treatment. Aside from the aforementioned pain crisis, the patient did not report any additional symptoms or side effects associated with crizanlizumab.

### Patient 2

Patient 2 is a 25-year-old African American man with a history of asthma, SCD (HbSS), and priapism (Table 1). The patient has a history of approximately 3 to 4 VOCs per year that required hospital admission and was receiving hydroxyurea for the management of SCD.

Patient 2 had his first experience with priapism when he was a child. Episodes became more frequent until the patient was having nightly episodes around 3:00 am that lasted <2 hours. Symptoms included a very sharp pain in the groin associated with a prolonged erection. Self-care was done by walking, taking a hot shower, or doing jumping jacks. These techniques had mixed results. The patient was once admitted to the hospital for a penile aspiration procedure due to a severe priapic event.

The patient received his first crizanlizumab infusion in October 2020 at a dose of 5 mg/kg, followed by an infusion 2 weeks later and every 4 weeks thereafter. After the first infusion, the patient experienced a decrease in the number of priapic events. Instead of occurring nightly, events occurred 3 to 4 times a week. By the third infusion, he reported almost never having priapic events, except during the 2 to 3 days before the next crizanlizumab infusion. During his infusions, he sometimes feels tired; however, the patient denies any serious symptoms or reactions. The patient qualitatively self-reported improved quality of life since initiating treatment.

### Patient 3

Patient 3 is a 39-year-old African American man with SCD (HbS/β^0^-thalassemia) and priapism (Table 1). The patient experienced 1 to 2 VOCs per year that required hospitalization. He experienced treatment failure with hydroxyurea, so it was discontinued.

The patient first experienced stuttering priapism in 2015 when he developed late-night to early-morning erections that were accompanied by groin discomfort and pain. Priapic events became more frequent until the patient had approximately 20 episodes a month, each lasting 30 minutes to 1 hour. Self-care included walking or taking a cold shower. These methods were often successful. After the patient’s initial episode, his urologist recommended a medication that could be injected into the penis. He does not recall the name of the medication and never initiated treatment because he was apprehensive about the procedure.

In April 2021, the patient started crizanlizumab at a dose of 5 mg/kg, with an infusion 2 weeks later and every 4 weeks thereafter. The patient experienced a substantial decrease in the number of priapic events and now reports only 4 events per month. The patient developed a pain episode during the second infusion, which was treated at the infusion center with IV fluids, ibuprofen, and acetaminophen. He is now premedicated with acetaminophen 30 minutes prior to infusions and has not had any more reactions. The patient qualitatively self-reported major improvement in his quality of life since initiating treatment.

## Discussion

No therapies or standardized treatments are currently approved to treat SCD-related priapism. Instead, patients are left to struggle with nightly events and to self-treat priapism at home. Hydroxyurea has been used to decrease priapic events in those with SCD. However, the overall effectiveness remains unknown, and there is no clear guidance on dosage.^3^ While events are generally self-limiting, home treatment does not prevent future attacks and may not decrease the duration of individual events.^1-3^ Repeated episodes of stuttering priapism can lead to erectile dysfunction, and prompt treatment is necessary to avoid permanent damage.^3^ Untreated priapism can also cause poor sleep quality, chronic pain, sexual dysfunction, and psychological struggles, resulting in major impairments to quality of life.^4^ Effective treatment options are needed to reduce the number of priapic events, decrease the risk of long-term complications, and improve quality of life.

All patients discussed in this case series experienced a decrease in the number of priapic events after starting crizanlizumab. One patient achieved a complete response and is no longer suffering from priapism, while the others showed a substantial decrease in the number of events. Two patients developed mild IRRs that were effectively managed, in 1 case with premedication. In clinical trials, IRRs were observed in approximately 3% of those receiving crizanlizumab infusions.^5^ In our experience, most IRRs respond quickly to medication, and supportive measures can be considered for subsequent infusions on a case-by-case basis. All patients reported improvements in quality of life due to the reduction in priapic events, which they attributed to crizanlizumab.

To our knowledge, these are the first published data on patients with SCD-related priapism who were successfully treated with crizanlizumab. Crizanlizumab is approved to reduce the frequency of VOCs in patients aged 16 years and older with SCD and is currently under investigation for the treatment of SCD-related priapism in the SPARTAN clinical trial (NCT03938454).^5,6^ The SPARTAN trial is currently recruiting, with results expected in 2023.^6^

Given the findings in these 3 patients, crizanlizumab shows promise as a potential agent in the management of SCD-related priapism.
